# Nutritional Composition, Micronutrient Fortification, and Processing Level of Plant-Based Yogurt Alternatives Available in Major Polish Retail Chains: A Retail Audit in Poznań, Poland

**DOI:** 10.3390/nu18111739

**Published:** 2026-05-28

**Authors:** Matylda Kręgielska-Narożna, Katarzyna Pastusiak-Zgolińska, Anna Mieczyńska, Agnieszka Seraszek-Jaros, Paweł Bogdański

**Affiliations:** 1 Department of Obesity and Metabolic Disorders Treatment and Clinical Dietetics, Poznan University of Medical Sciences, 60-355 Poznań, Poland; kpastusiak@ump.edu.pl (K.P.-Z.); pbogdanski@ump.edu.pl (P.B.); 2 Student Scientific Society, Poznan University of Medical Sciences, 60-355 Poznań, Poland; 89162@student.ump.edu.pl; 3 Department of Bioinformatics and Computational Biology, Poznan University of Medical Sciences, 60-355 Poznań, Poland; seraszek@ump.edu.pl

**Keywords:** plant-based yogurt alternatives, dairy alternatives, plant-based diet, micronutrient fortification, ultra-processed foods, retail audit, nutritional composition

## Abstract

**Background**: The market for plant-based yogurt alternatives has rapidly expanded, reflecting the growing popularity of plant-based diets. However, their nutritional profiles and micronutrient fortification often differ substantially from those of traditional dairy yogurt. **Methods**: This study conducted a cross-sectional audit of retail labels on fermented plant-based yogurt alternatives available in major Polish retail chains. Data were collected in 2024 from eight stores across four nationwide supermarket chains. Nutritional composition, primary plant ingredient, micronutrient fortification, and processing level (NOVA classification) were recorded from product labels, while nutrient values were summarized using descriptive statistics and compared across product categories. **Results**: A total of 62 plant-based yogurt alternatives were identified, including 49 fruit-flavored and 13 natural products. Coconut was the predominant plant ingredient (54.8%), followed by soy (24.2%) and oat (11.3%). Fruit-flavored products contained significantly higher carbohydrate and sugar levels than natural ones. Soy-based products exhibited the highest protein content, often approaching that of conventional dairy yogurt, whereas coconut-based products were characterized by the lowest protein and higher saturated fat content. Overall, 37.1% of products were fortified with at least one micronutrient, primarily calcium, vitamin D, and vitamin B12. Most products were classified as ultra-processed (NOVA 4). **Conclusions**: Plant-based yogurt alternatives available on the Polish market are nutritionally diverse. Their composition is heavily influenced by the primary plant ingredient and fortification practices. Many of these products cannot be considered direct nutritional equivalents to dairy yogurt, underscoring the need for careful formulation, effective micronutrient fortification, and transparent labeling.

## 1. Introduction

Milk and dairy products provide essential nutrients and are associated with favorable outcomes for bone health and reduced risks of various noncommunicable diseases when fermented [1,2,3,4,5]. Nevertheless, environmental and sustainable nutrition guidelines encourage limiting animal-based product consumption, especially in high-consumption contexts, while promoting increased intake of plant-based foods [6]. This shift is evident in consumer behavior and market availability, particularly in Poland, where the sales and availability of plant-based foods—specifically plant-based substitutes for milk and fermented dairy—have surged [7,8].

Plant-based diets are linked to lower risks of insulin resistance and cardiometabolic diseases, potential reductions in certain cancer risks, and support for weight management when well planned [9,10]. Notably, studies indicate that merely increasing plant intake—without considering nutrient density—may not guarantee adequate intakes of key nutrients. This is particularly crucial for dairy replacements, especially concerning high-quality protein, calcium, and vitamin B12 [11]. Motivations for selecting plant-based products vary—health, ethics, cost, and taste constitute factors, and the climate benefits of adopting plant-rich diets are increasingly recognized [12,13].

Plant-based dairy analogues exhibit significant variability in nutritional quality and sensory characteristics. Several studies have documented lower protein content and higher levels of certain additives in some categories of plant-based alternatives than in their traditional dairy counterparts, with notable differences based on primary plant ingredient (e.g., soy, coconut, and oat) and product type (plain vs. fruit-flavored). Additionally, these alternatives often come at higher prices, potentially impacting affordability and equitable access [14,15,16]. Despite rapid market expansion, systematic retail-level assessments of fermented plant-based yogurt alternatives in Central and Eastern Europe remain limited, particularly regarding micronutrient fortification and nutritional comparability to dairy yogurt. Therefore, this study specifically examined fermented plant-based “yogurt-like” products, focusing on fruit-flavored variants available in major Polish retail chains. We analyzed their nutrient composition, fortification practices, and processing markers, interpreting nutrient values in relation to conventional dairy yogurt reference data from national food composition tables. Using this dairy benchmarking approach, the study aimed to assess the nutritional quality of plant-based yogurt alternatives available in Poland (Poznań) and provide evidence relevant to patient education and healthy, sustainable dietary choices.

## 2. Materials and Methods

### 2.1. Study Design and Setting

This cross-sectional retail label audit focused on fermented plant-based yogurt alternatives. Data were collected in January and March 2024 in Poznań, Poland. Four major nationwide retail chains—Auchan, Lidl, Biedronka, and Kaufland—were selected based on their leading market position and consumer reach, as documented in national retail market reports [17]. Two stores were randomly selected from each chain, resulting in a total of eight audited stores. All eligible products available during visits were recorded, and the same stores were revisited in March 2024. Product assortment remained consistent between both visits, indicating short-term stability in product availability. Considering that these chains operate nationwide with largely standardized offerings, the audited products are broadly representative of the national market, allowing us to treat products identified in both visits as a single analytical sample.

### 2.2. Product Selection

Eligible products were defined as plant-based yogurt alternatives intended to substitute traditional dairy yogurt and sold in the refrigerated section. Products were included if they (1) were marketed as yogurt-style or “yogurt-like,” (2) required refrigeration at the point of sale, (3) contained no dairy-derived ingredients, and (4) listed live starter cultures on the label. They were categorized into two groups based on flavor: (1) natural/plain and (2) fruit-flavored or flavored variants. Products were excluded if they were shelf-stable, plant-based desserts not marketed as yogurt alternatives, beverages, or products containing any dairy ingredients (e.g., milk proteins, lactose, whey, etc.). All eligible products available during data collection were recorded.

### 2.3. Data Collection and Quality Control

Data collection occurred in two waves—January and March 2024—across the same selected retail stores. In each wave, a trained researcher visited each store and systematically reviewed the refrigerated section for plant-based yogurt alternatives. All eligible products were photographed in-store, capturing the front-of-pack information, nutrition declaration, ingredient list, and any voluntary micronutrient declarations. The data were subsequently transcribed into a structured spreadsheet developed specifically for this study, based on typical product packaging information, including nutrient values per 100 g, ingredient lists, fortification declarations, and selected product claims. Researchers received standardized instructions prior to data collection to ensure consistent identification of eligible products and uniform data recording. The March data collection served as a verification step, where products recorded in January were rechecked, and the transcribed data were reviewed for accuracy. No changes in product availability, formulation, or nutrient composition were observed between the two waves, enabling the dataset to be treated as a single cross-sectional snapshot of the market in early 2024.

### 2.4. Variables

The following variables were extracted from product labels and recorded per 100 g:

- Nutritional composition: Energy (kJ and kcal), total fat (g), saturated fat (g), carbohydrates (g), sugars (g), protein (g), fiber (g), and salt (g).
- Micronutrients (if declared): Calcium (mg), vitamin B12 (µg), vitamin D (µg), riboflavin (vitamin B2, mg), vitamin E (mg), and iodine (µg). Micronutrient values were recorded only when quantitative information was provided.
- Product characteristics: Flavor category (natural/plain vs. flavored) and primary plant ingredient (soy, coconut, oat, cashew, and fava bean), defined as the first plant-derived ingredient listed on the ingredient list. For products containing multiple plant ingredients, classification was based on the predominant ingredient, as indicated by its first position on the label.
- Fortification: Products were classified as fortified (yes/no) if calcium, vitamin D, vitamin B12, riboflavin (vitamin B2), vitamin E, or iodine were declared as added ingredients.
- Processing level: Products were classified according to the NOVA system based on the ingredient list and the presence of additives or technological ingredients.

### 2.5. NOVA Classification

Products were classified according to the NOVA food classification system, which categorizes foods based on the extent and purpose of industrial processing [18]. This classification was based on the ingredient list provided on product packaging and predefined NOVA criteria.

### 2.6. Benchmarking Against Dairy Yogurt

Reference values were derived from Polish food composition tables [19]. Plain (2% fat) and strawberry (1.5% fat) yogurt were selected as representative traditional dairy yogurt products commonly available in Poland. Benchmark values per 100 g were extracted for energy, total fat, saturated fat, carbohydrates, sugars, protein, calcium, vitamin B12, and vitamin D, serving as contextual reference points for interpreting the nutritional composition of plant-based yogurt alternatives.

No inferential statistical comparisons were made between plant-based products and reference dairy yogurt values. Instead, benchmark values were utilized to indicate the proportion of products that satisfied or approximated nutrient levels typical of conventional dairy yogurt.

### 2.7. Statistical Analysis

All statistical analyses were performed using Statistica software (TIBCO Software Inc., Palo Alto, CA, USA, 2017), version 13 (https://www.tibco.com accessed on 28 May 2026). Normality of distribution for continuous variables was assessed using the Shapiro–Wilk test. Since none of the analysed variables met the assumption of normality, all analyses were conducted using non-parametric tests. Continuous variables are presented as medians with interquartile ranges (Q1–Q3). Differences between two independent groups (natural vs. fruit-flavored products) were assessed using the Mann–Whitney U test. For comparisons involving more than two plant-based product categories (soy-, coconut- and oat-based products), the Kruskal–Wallis test was applied. For variables that showed statistically significant differences in the Kruskal–Wallis test, post hoc pairwise comparisons were performed using the Dunn test with Bonferroni correction. Given three pairwise comparisons, the adjusted significance level was set at α = 0.05/3 = 0.0167. All differences that were significant in the global test remained significant after Bonferroni correction. For variables with a non-significant Kruskal–Wallis result (e.g., sugars), no post hoc testing was conducted.

Categorical variables were analysed using the chi-square test. A *p*-value below the adopted significance threshold (*p* < 0.05, or *p* < 0.0167 for Bonferroni-adjusted post hoc comparisons) was considered statistically significant.

## 3. Results

### 3.1. Product Characteristics

A total of 62 fermented plant-based yogurt alternatives were identified, comprising 49 (79.0%) fruit-flavored and 13 (21.0%) natural/plain products. Coconut was the most prevalent primary plant ingredient (54.8%), followed by soy (24.2%) and oat (11.3%). Additionally, 37.1% of products were fortified with at least one micronutrient, and most were classified as NOVA 4 (Table 1).

### 3.2. Nutritional Composition

The nutritional composition of natural and fruit-flavored plant-based yogurt alternatives is detailed in Table 2. Fruit-flavored products contained significantly higher carbohydrate and sugar contents than natural variants (12.9 vs. 5.9 g/100 g and 7.6 vs. 0.6 g/100 g, respectively; *p* < 0.0001). Conversely, natural products exhibited a higher total fat content (5.3 vs. 4.2 g/100 g; *p* = 0.036). No other significant differences were noted.

### 3.3. Fortification Practices

In total, 23 of the 62 products (37.1%) were fortified with at least one micronutrient. Fortification was present in 30.8% and 38.8% of natural and fruit-flavored products, respectively. The prevalence of individual micronutrients is summarized in Table 3. The declared added micronutrients were as follows: calcium, vitamin B12, vitamin D, riboflavin (vitamin B2), vitamin E, and iodine. Calcium was the most frequently added micronutrient, followed by vitamin D and vitamin B12, while riboflavin, iodine, and vitamin E were less frequently included.

### 3.4. Differences by Plant Ingredient

Differences according to the primary plant ingredient were examined among fruit-flavored products only, as the number of natural products within each category was insufficient for meaningful inferential comparisons. Therefore, our analysis focused on coconut-, soy-, and oat-based fruit-flavored products owing to adequate group sizes.

Significant differences were noted across plant bases for energy, total fat, saturated fat, carbohydrates, protein, fiber, and salt content (*p* < 0.05), with no significant differences observed for sugars. Soy-based products yielded the highest median protein content (3.7 g/100 g), significantly exceeding coconut- and oat-based alternatives. In contrast, coconut-based products exhibited markedly higher total fat, saturated fat, and energy values than soy-based products. Oat-based products displayed higher carbohydrate content than soy-based products, while soy-based products also exhibited higher fiber and salt content. Detailed results are presented in Table 4.

### 3.5. Benchmark Context

The proportion of products meeting selected nutritional benchmarks varied considerably across nutrients and product types.

**Figure 1:**
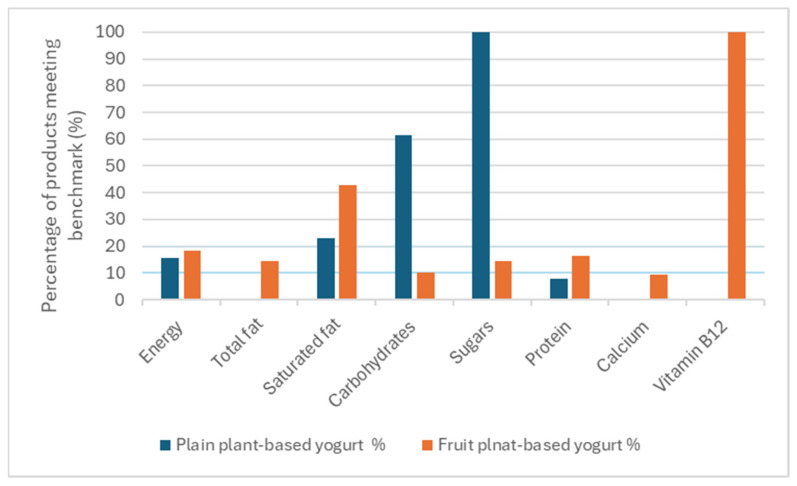
Percentage of plant-based yogurt alternatives meeting selected nutritional benchmarks by product type (in plain and fruit-flavored groups).

Among natural plant-based yogurt alternatives, compliance with several benchmarks was limited. None of the products satisfied the total fat or calcium benchmarks, and only one product (7.7%) fulfilled the protein benchmark typical of conventional plain yogurt. Conversely, all natural products met the sugar benchmark, and a larger proportion satisfied the carbohydrate benchmark (61.5%) (Table 5, Figure 1).

**Figure 2:**
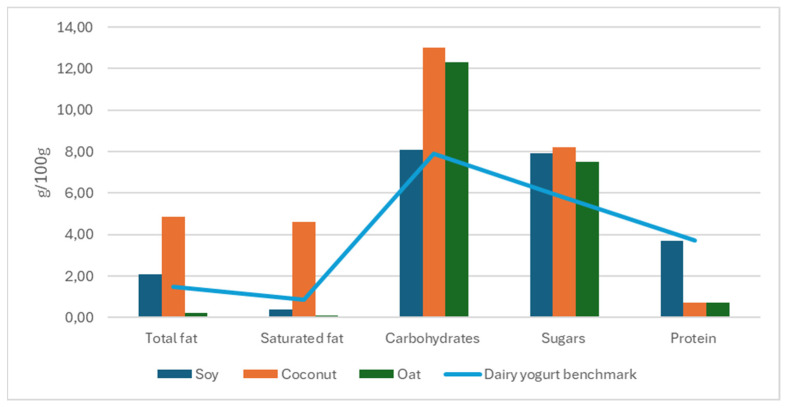
Median macronutrient content of fruit-flavored plant-based yogurt alternatives by primary plant ingredient, compared with benchmark values derived from conventional dairy yogurt. Benchmark shown for contextual reference.

For fruit-flavored products, compliance with the protein benchmark was relatively low (16.3%). Only a small proportion fulfilled the benchmarks for total fat (14.3%) and sugars (14.3%), while 42.9% satisfied the saturated fat benchmark (Table 6, Figure 1). These patterns reflect differences in median macronutrient content across primary plant ingredients, as illustrated in Figure 2. Compliance with selected benchmarks varied significantly based on the primary plant ingredient: soy-based products predominantly met the protein benchmark, whereas coconut-based products generally contained lower protein levels but often exceeded the saturated fat thresholds. Table 7 additionally presents the relative median nutrient values in relation to benchmark dairy yogurt values among fruit-flavored soy- and coconut-based yogurt alternatives.

## 4. Discussion

Recent evidence suggests that the market for plant-based yogurt alternatives is rapidly expanding, reflecting a broader transition towards plant-based diets in numerous countries [20]. The increasing popularity of these diets may offer environmental and health benefits [21,22]. However, the nutritional quality of plant-based foods varies significantly, and plant-based yogurt alternatives should therefore not be treated as nutritionally homogeneous products, which has important implications for dietary assessment and consumer guidance. Our findings emphasize that this heterogeneity is not merely a technical detail but a significant factor that could influence the overall nutritional value of the consumer’s diet. Plant-based alternatives do not provide direct, one-to-one nutritional equivalents to animal-based products [23,24]. While some consumers perceive plant-based dairy alternatives as substitutes for conventional dairy products, research indicates that these products are often regarded as distinct categories [25,26]. The nutritional composition of plant-based alternatives can differ widely, both across the category and among individual products, depending on formulation and the primary plant ingredient [27,28,29].

A key finding of this study is that the primary plant ingredient significantly influences the macronutrient composition and overall nutritional value of plant-based yogurt alternatives. Notably, soy-based products exhibited considerably higher protein levels than coconut-based options, frequently approaching the benchmark protein levels found in conventional dairy yogurt. This suggests that soy remains the most nutritionally viable base for those seeking a protein-equivalent replacement for dairy. Previous research supports this trend, indicating that soy alternatives typically contain more protein than those prepared from coconut or other plant sources [30,31]. Protein is an essential nutrient, and adequate intake plays an indispensable role in maintaining overall nutritional balance [32]. In contrast, coconut-based yogurt alternatives—when used as substitutes for dairy yogurt—tend to offer relatively lower protein levels while often being higher in total fat and saturated fatty acids [31,33]. Considering that coconut was the predominant base in our study (54.8%), this market dominance may pose a public health challenge, as consumers may inadvertently increase their intake of saturated fats—contrary to cardiovascular health guidelines—while missing out on essential protein [34,35]. Additionally, the wide variability observed for certain nutrients, particularly among oat-based products, likely reflects differences in product formulations across brands. From a nutritional perspective, these differences may be particularly relevant for individuals relying on plant-based diets, where plant-based yogurt alternatives can be perceived as direct substitutes for dairy products.

The study also established that sugar content varied primarily with flavor variants; fruit-flavored products yielded higher carbohydrate and sugar levels than their natural counterparts. This aligns with previous research, which attributes the increased carbohydrate content to added fruit preparations and flavoring ingredients [36]. The stark contrast between natural (0.6 g/100 g) and fruit-flavored (7.6 g/100 g) products highlights that the “health halo” surrounding plant-based diets can be compromised by excessive added sugars in popular flavored variants. This finding is relevant in the context of dietary recommendations aimed at reducing free sugar intake, particularly in populations with high consumption of flavored dairy alternatives [37,38,39].

Micronutrient fortification represents another vital consideration for plant-based dairy alternatives. In this analysis, soy-based products were most commonly fortified, while numerous products available on the market lacked additional micronutrients. The observation that 62.9% of products were not fortified with any micronutrients suggests a significant gap in the industry’s commitment to nutritional parity with dairy. Research emphasizes the importance of fortification in plant-based dairy alternatives to ensure adequate intake of nutrients such as calcium and vitamin B12, which are naturally present in dairy products but often lacking or deficient in plant-based foods [40,41]. Even among fortified products in this study, few matched the nutrient levels of conventional dairy yogurt, particularly for key nutrients such as calcium and vitamin B12. Furthermore, the lack of mandatory micronutrient declarations unless linked to health claims (REGULATION (EU) No 1169/2011) [42] creates a “nutritional lottery” for consumers, who cannot reliably assess the micronutrient density of many products. The inconsistency in fortification practices may limit the ability of plant-based yogurt alternatives to serve as reliable sources of key micronutrients, particularly in populations at risk of deficiencies.

Another relevant aspect of plant-based yogurt alternatives is their level of processing. In this analysis, most products were classified as NOVA 4, indicating extensive industrial processing. This finding reveals a paradox: while plant-based diets are generally associated with lower risk of chronic diseases, the current market for fermented alternatives is dominated by ultra-processed foods (UPFs), which are linked to adverse health outcomes in several epidemiological studies [43,44,45]. However, the high processing level in plant-based yogurt alternatives may be necessary to achieve the desired texture, stability, and sensory attributes. This highlights a potential conflict between technological functionality and nutritional quality, which may have important health implications, particularly in the context of the increasing consumption of ultra-processed plant-based foods and their potential impact on the risk of chronic diseases. Remarkably, some oat-based products exhibited lower processing levels. Moreover, conventional dairy products can also vary in their NOVA classifications based on formulation and processing extent.

Taken together, these findings suggest that while plant-based yogurt alternatives may support dietary shifts towards more sustainable eating patterns, their nutritional quality remains highly variable and should be critically evaluated in both clinical practice and public health recommendations. Dietary guidelines and patient education must move beyond treating this category as a uniform substitute for dairy, instead focusing on the critical reading of labels to identify protein-rich and fortified options.

This study has certain limitations. It was conducted as a retail shelf audit in a single city, which may not fully reflect the diversity of products available across the entire country.

However, since the selected retail chains (Auchan, Lidl, Biedronka, Kaufland) operate nationwide with largely standardized assortments and a high proportion of private-label products, these results can be considered representative of the broader Polish retail landscape.

In addition, nutrient values derived from labels may differ slightly from analytically measured compositions. Nonetheless, the findings offer valuable insights into the current composition of plant-based yogurt alternatives available in Poland. Overall, the results reveal significant heterogeneity in this product category, with nutritional composition strongly influenced by the primary plant ingredient.

Future research should include a broader geographic scope and a more diverse range of retail settings to further validate these findings. A more comprehensive understanding of plant-based yogurt alternatives could also be supported by incorporating aspects related to consumer behavior and perception.

## 5. Conclusions

Plant-based yogurt alternatives available in major retail chains in Poznań, Poland, demonstrate considerable nutritional diversity and variability in composition. The primary plant ingredient appears to be a key determinant of the macronutrient profile, especially protein and fat content. Soy-based products generally contain higher protein levels and more frequently satisfy nutritional benchmarks set by conventional dairy yogurt, while coconut-based options typically provide less protein and more saturated fat. Most products are classified as ultra-processed, indicating a high level of industrial processing, which may further impact their overall nutritional profile and health implications.

In several instances, plant-based yogurt alternatives do not serve as direct nutritional equivalents to dairy yogurt, particularly regarding protein, calcium, and vitamin B12. Therefore, appropriate micronutrient fortification and careful product formulation are essential for enhancing the nutritional value of plant-based products as they gain popularity. These findings highlight the importance of transparent labeling, effective fortification, and reliable nutritional information to support informed consumer choices and evidence-based dietary recommendations. In light of current global challenges, food systems are increasingly expected to promote both public health and environmental sustainability.

## Figures and Tables

**Table 1:** Characteristics of fermented plant-based yogurt alternatives.

Characteristics	Total (*n* = 62)	Fruit (*n* = 49)	Natural (*n* = 13)
Product type			
Fruit-flavored	49 (79.0%)	49 (100%)	0 (0%)
Natural	13 (21.0%)	0 (0%)	13 (100%)
Primary plant ingredient			
Coconut	34 (54.8%)	24 (49.0%)	10 (76.9%)
Soy	15 (24.2%)	13 (26.5%)	2 (15.4%)
Oat	7 (11.3%)	7 (14.3%)	0 (0%)
Fava bean	3 (4.8%)	3 (6.1%)	0 (0%)
Cashew	3 (4.8%)	2 (4.1%)	1 (7.7%)
Fortification (≥1 micronutrient)			
Fortified	23 (37.1%)	19 (38.8%)	4 (30.8%)
Not fortified	39 (62.9%)	30 (61.2%)	9 (69.2%)
NOVA classification			
NOVA 1	4 (6.5%)	4 (8.2%)	0 (0%)
NOVA 4	58 (93.5%)	45 (91.8%)	13 (100%)

**Table 2:** Nutritional composition of natural and fruit-flavored plant-based yogurt alternatives.

Variable	Natural (*n* = 13), Median (Q1–Q3)	Fruit-Flavored (*n* = 49), Median (Q1–Q3)	*p*-Value
Energy (kJ)	362 (283–387)	382 (279–431)	0.382
Energy (kcal)	87 (68–93)	91 (66–103)	0.451
Total fat (g)	5.3 (3.7–7.1)	4.2 (2.1–5.3)	0.036
Saturated fat (g)	5.0 (3.3–6.8)	3.9 (0.4–5.1)	0.061
Carbohydrates (g)	5.9 (4.9–6.6)	12.9 (9.8–13.4)	<0.0001
Sugars (g)	0.6 (0.5–0.9)	7.6 (7.0–8.4)	<0.0001
Protein (g)	0.9 (0.7–1.0)	0.9 (0.7–3.2)	0.986
Fiber (g)	0.5 (0.5–1.5)	0.5 (0.5–0.9)	0.786
Salt (g)	0.03 (0.02–0.07)	0.04 (0.02–0.16)	0.515

**Table 3:** Prevalence of micronutrient fortification in plant-based yogurt alternatives.

Micronutrient	Total (*n* = 62), *n* (%)	Fruit-Flavored (*n* = 49), *n* (%)	Natural (*n* = 13), *n* (%)
Calcium	25 (40.3%)	21 (42.9%)	4 (30.8%)
Vitamin D	24 (38.7%)	21 (42.9%)	3 (23.1%)
Vitamin B12	22 (35.5%)	19 (38.8%)	3 (23.1%)
Riboflavin (B2)	13 (21.0%)	13 (26.5%)	0 (0%)
Vitamin E	1 (1.6%)	1 (2.0%)	0 (0%)
Iodine	3 (4.8%)	3 (6.1%)	0 (0%)

**Table 4:** Nutritional composition of fruit-flavored plant-based yogurt alternatives by primary plant ingredient.

Variable (per 100 g)	Soy (*n* = 13), Median (Q1–Q3)	Coconut (*n* = 24), Median (Q1–Q3)	Oat (*n* = 7), Median (Q1–Q3)	*p*-Value
Energy (kcal)	69 (62–72)	100.5 (94–113)	57 (43–185)	0.0001
Total fat (g)	2.1 (2.0–2.2)	4.9 (4.3–6.7)	0.2 (0.2–11.0)	<0.0001
Saturated fat (g)	0.4 (0.4–0.4)	4.6 (4.0–6.4)	0.1 (0.1–8.0)	<0.0001
Carbohydrates (g)	8.1 (5.7–9.8)	13.0 (13.0–14.0)	12.3 (9.2–18.0)	<0.0001
Sugars (g)	7.9 (4.8–8.2)	8.2 (7.0–8.6)	7.5 (5.5–14.0)	0.421
Protein (g)	3.7 (3.6–3.8)	0.7 (0.6–0.8)	0.7 (0.5–3.0)	<0.0001
Fiber (g)	0.9 (0.8–1.1)	0.5 (0.5–0.5)	0.9 (0.7–1.0)	<0.0001
Salt (g)	0.22 (0.20–0.25)	0.04 (0.03–0.05)	0.02 (0.01–0.10)	<0.0001

For the variables presented in the table, post hoc comparisons were performed using the Bonferroni correction, and statistical significance was set at *p* = 0.0167.

**Table 5:** Comparison of natural plant-based yogurt alternatives with nutritional benchmarks derived from conventional plain yogurt (2% fat).

Nutrient (per 100 g)	Benchmark Value	Products Satisfying Benchmark, *n* (%)			
		All (*n* = 13)	Soy (*n* = 2)	Coconut (*n* = 10)	Cashew (*n* = 1)
Energy (kcal)	60	2 (15.4%)	0	1	1
Total fat (g)	≤2	0 (0%)	0	0	0
Saturated fat (g)	≤1.19	3 (23.1%)	2	0	1
Carbohydrates (g)	6.2	8 (61.5%)	0	8	0
Sugars (g)	≤3.2	13 (100%)	2	10	1
Protein (g)	≥4.3	1 (7.7%)	1	0	0
Calcium (mg) *	≥170	0 (0%)	0	0	0
Vitamin B12 (μg) *	≥0.5	0 (0%)	0	0	0

* Calculated among products declaring the respective micronutrient content on the label.

**Table 6:** Comparison of fruit-flavored plant-based yogurt alternatives with nutritional benchmarks derived from conventional strawberry yogurt (1.5% fat).

Nutrient (per 100 g)	Benchmark Value	Products Satisfying Benchmark *n* (%)					
		All (*n* = 49)	Soy (*n* = 13)	Coconut (*n* = 24)	Oat (*n* = 7)	Fava bean (*n* = 3)	Cashew (*n* = 2)
Energy (kcal)	61	9 (18.4%)	5	0	1	3	0
Total fat (g)	≤1.5	7 (14.3%)	0	0	4	3	0
Saturated fat (g)	≤0.88	21 (42.9%)	12	0	4	3	2
Carbohydrates (g)	7.9	5 (10.2%)	4	0	1	0	0
Sugars (g)	≤5.8	7 (14.3%)	5	0	2	0	0
Protein (g)	≥3.7	8 (16.3%)	8	0	0	0	0
Calcium (mg) *	≥134	2 (9.5%)	0	2	0	0	0
Vitamin B12 (μg) *	≥0.35	19 (100%)	13	3	3	0	0

* Calculated among products declaring the respective micronutrient content on the label.

**Table 7:** Compliance of fruit-flavored soy- and coconut-based yogurt alternatives with benchmark nutrient values (%).

Primary Plant Base	Soy		Coconut	
Nutrient (per 100 g)	Median relative to benchmark (%)	Interpretation	Median relative to benchmark (%)	Interpretation
Total fat (g)	140%	Higher than benchmark	323.3%	Substantially higher
Saturated fat (g)	45.5%	Substantially lower	522.7%	Substantially higher
Carbohydrates (g)	102.5%	Comparable to benchmark	164.6%	Higher than benchmark
Sugars (g)	136.2%	Higher than benchmark	141.4%	Higher than benchmark
Protein (g)	100%	Comparable to benchmark	18.9%	Substantially lower

Relative values were calculated using the formula: median nutrient value/benchmark value × 100%. Values between 90–110% were considered nutritionally comparable to the benchmark; values below 90% were interpreted as lower, whereas values above 110% were interpreted as higher than the benchmark.

## Data Availability

The data supporting the findings of this study are presented in the article.
